# Sodium-Glucose Cotransporter-2 Inhibitor versus Beta-Blocker Use for Hepatocellular Carcinoma Risk among People with Hepatitis B or C Virus Infection and Diabetes Mellitus

**DOI:** 10.3390/cancers15072104

**Published:** 2023-03-31

**Authors:** Wei-Syun Hu, Cheng-Li Lin

**Affiliations:** 1 School of Medicine, College of Medicine, China Medical University, Taichung 40402, Taiwan; 2 Division of Cardiovascular Medicine, Department of Medicine, China Medical University Hospital, Taichung 40447, Taiwan; 3 Management Office for Health Data, China Medical University Hospital, Taichung 40447, Taiwan

**Keywords:** beta-blockers, hepatitis B, hepatitis C, hepatocellular carcinoma, sodium-glucose cotransporter-2 inhibitor

## Abstract

**Simple Summary:**

The authors used a large Taiwanese database of patients with chronic hepatitis B or C in order to study if SGLT2I, as compared to BB, may decrease HCC. In brief, SGLT2I caused a risk reduction in the likelihood of HCC development of about 73%.

**Abstract:**

**Objective:** The current study detects the effect of sodium-glucose cotransporter-2 inhibitor (SGLT2I) versus beta-blocker (BB) in diabetes mellitus (DM) with chronic hepatitis B or C on hepatocellular carcinoma (HCC) outcomes. **Methods:** The multivariate logistic regression model, including all baseline characteristics and index year, was used to calculate the propensity scores, and we performed the greedy algorithm on propensity scores to create matched pairs of SGLT2I and BB users. Hazard ratios (HRs) and the corresponding 95% confidence intervals (CIs) of HCC were estimated by Cox proportional hazards regression models, and we adjusted for confounding factors by including the baseline characteristics in the regression models. **Results:** After matching in a ratio of 1:1, 7023 SGLT2I users and 7023 BB users were included in the following statistical analyses. The overall HRs showed a significantly lower risk of HCC in SGLT2I users in comparison to a reference group of BB users with an adjusted HR of 0.27 (0.21, 0.34). **Conclusions:** Compared to BB use, SGLT2I was associated with a significant risk reduction in HCC occurrence.

## 1. Introduction

The burden of hepatocellular carcinoma (HCC) on chronic hepatitis B or C is obvious [1,2]. While chronic hepatitis B or C coexists with diabetes mellitus (DM), the risk of developing HCC is significantly elevated [3,4]. Beta-blockers (BB) are used for the treatment/prophylaxis of variceal bleeding in portal hypertension, and this is an obvious confounder of why there is an apparent association between beta-blocker use and worse liver disease. The use of beta-blockers as a control group for SGLT2I might be reasonable, although the use of BB for cancer prevention for people affected by chronic hepatitis B or C is not recognized worldwide [5,6]. Recently, the widespread use of sodium-glucose cotransporter-2 inhibitor (SGLT2I) was shown due to several pleiotropic phenomena in addition to a pure glucose-lowering effect, suggestive of non-cardiovascular risk reduction in SGLT2I [7,8]. There seems to be clinical utility in examining the association of SGLT2I for HCC among DM + chronic hepatitis B or C. Hence, this retrospective study investigated the effect of SGLT2I versus BB in DM with chronic B or C on HCC outcomes. 

## 2. Methods

### 2.1. National Health Insurance

The National Health Insurance (NHI) program in Taiwan is a nationwide healthcare system established on 1 March 1995 and covers ~99% of the population in Taiwan [9]. The healthcare reimbursements submitted to the NHI administration include information regarding demographics, diagnoses of diseases, which were made based on the ICD-9-CM and ICD-10-CM codes, prescriptions of medications, and dates of clinic visits or hospitalizations. The database was used in this retrospective cohort study, and the data were encrypted for privacy preservation. The academic research and waivers of informed consent were approved by the Research Ethics Committee of the China Medical University and the Hospital in Taichung, Taiwan (CMUH110-REC1-038(CR-2)).

### 2.2. Study Population

The diseases and medications used in the study were defined and summarized in Table S1. The study included 111,865 patients with DM and HBV/HCV. A total of 31,215 patients received SGLT2I after the last date of the first diagnoses of DM and HBV/HCV, and 80,650 patients received BB after the same date as above. The first date of the prescription was defined as the index date. The end of the follow-up period was the new onset of HCC, death, or 31 December 2019. The exclusion criteria were as follows: patients receiving both SGLT2I and BB during the observation period (*n* = 7686); patients diagnosed with HCC before or at the index date (*n* = 8389); patients aged less than 20 years (*n* = 28); patients without valid sex categories (*n* = 78); and index dates not between 2016 and 2018 (*n* = 62,728). As a result, 14,313 SGLT2I users and 18,643 BB users were identified. To reduce differences between baseline characteristics of the two groups, propensity scores were used. Baseline characteristics considered in the study included sex, age, hyperlipidemia, hypertension, obesity, coronary heart disease, chronic obstructive pulmonary disease, chronic kidney disease, chronic liver disease and cirrhosis, alcohol-related disorders, α-glucosidase inhibitors, biguanides, dipeptidyl peptidase-4 inhibitors, meglitinides, sulphonylureas, thiazolidinediones, glucagon-like peptide-1 receptor agonists, and insulins. After matching in a ratio of 1:1, 7023 SGLT2I users and 7023 BB users were included in the following statistical analyses.

### 2.3. Statistical Analysis

SAS statistical software, version 9.4 (SAS Institute, Cary, NC, USA), was used to conduct the statistical analyses. We analyzed the data using two-tailed tests, and a *p*-value less than 0.05 is statistically significant. The multivariate logistic regression model, including all baseline characteristics, index year was used to calculate the propensity scores, and we applied the greedy algorithm to propensity scores to create matched pairs of SGLT2I and BB users. Statistical differences between baseline characteristics of the two groups were examined by Chi-square tests and independent *t*-tests. The cumulative days’ supplies of SGLT2I and BB were calculated for each user, and we categorized SGLT2I and BB users into two subgroups, respectively, based on the medians of the cumulative days’ supplies to explore the dose–response relationship. The incidence density rate (IR) of HCC was determined by the number of new onsets of HCC divided by the sum of person-years of the at-risk population. Hazard ratios (HRs) and the corresponding 95% confidence intervals (CIs) of HCC were estimated by Cox proportional hazards regression models, and we adjusted for confounding factors by including the baseline characteristics in the regression models. We plotted the Kaplan–Meier cumulative incidence of HCC for SGLT2I and BB users over time, and the differences between the two curves were tested by the log-rank test.

## 3. Results

**Figure 1:**
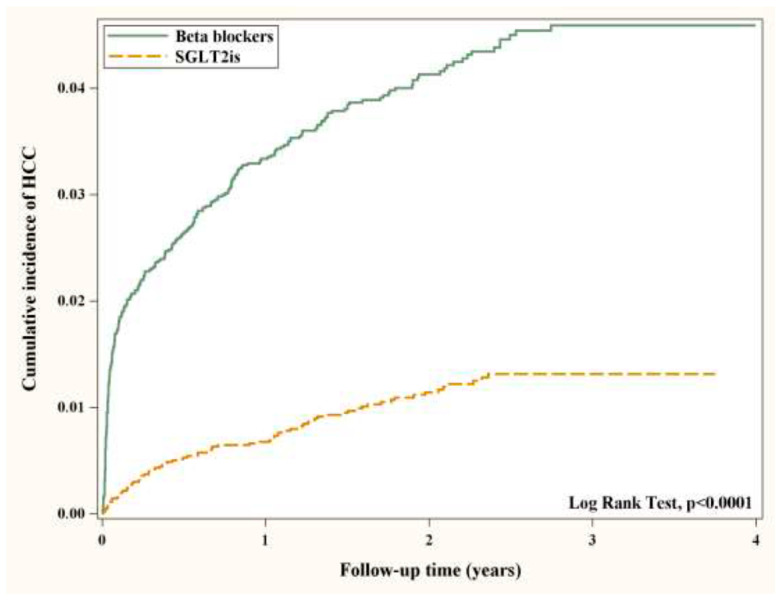
Cumulative incidence of HCC in SGLT2I and β-blocker users.

The baseline characteristics among DM patients with HBV/HCV receiving β-blockers or SGLT2is and the comparisons of the baseline characteristics between the two groups are listed in Table 1. After matching, no differences were observed in baseline characteristics between the two groups, suggesting that SGLT2i users were well matched with β-blocker users on the baseline characteristics. About 57% of the patients were male, and approximately 53% of them were aged more than 60 years old. Most of the patients were diagnosed with hyperlipidemia (~72%), hypertension (~73%), and chronic liver disease and cirrhosis (~65%). In Table 2, the overall HRs showed a significantly lower risk of HCC in SGLT2i users when compared to a reference group of β-blocker users (adjusted HR = 0.27 with 95% CI = [0.21, 0.34] for all). In Table 3, more SGLT2i use was significantly associated with a decreased risk of HCC (adjusted HR = 0.53 with 95% CI = [0.41, 0.68] for 1–532 days of use duration; adjusted HR = 0.04 with 95% CI = [0.02, 0.08] for >532 days of use duration); however, β-blocker users had a higher risk of HCC than SGLT2i users (adjusted HR = 4.20 with 95% CI = [3.20, 5.51] for 1–35 days of use duration; adjusted HR = 3.36 with 95% CI = [2.54, 4.45] for >35 days of use duration). Table 4 shows the IRs of HCC in β-blocker and SGLT2i users stratified by different types of hepatitis and the HRs along with the corresponding 95% CI in SGLT2i users compared to β-blocker users. Whichever hepatitis DM patients had, patients receiving SGLT2is were less likely to develop HCC in contrast to patients receiving β-blockers (adjusted HR = 0.25 with 95% CI = [0.18, 0.35] for HBV patients; adjusted HR = 0.32 with 95% CI = [0.22, 0.46] for HCV patients). Figure 1 shows the cumulative incidence of HCC between SGLT2i and β-blocker users. SGLT2i users had a significantly lower risk of HCC in contrast to β-blocker users (log-rank test *p* < 0.0001). 

## 4. Discussion

The authors used a large Taiwanese database of patients with chronic hepatitis B or C in order to study if SGLT2I, as compared to BB, may decrease HCC. They propensity-matched about 7023 patients in one group with BB and a similar-sized group with SGLT2I. In brief, even after controlling for some dissimilarities between the two groups, SGLT2I caused a risk reduction in the likelihood of HCC development of about 73%. 

The authors have detected a potential positive effect of the treatment with SGLT2I. The methodology on how HCC were defined and detected is also described, and matching with PSM was used [10,11,12]. This is a work reporting on a possible association between the use of SGLT2I and decreased incidence of HCC among people with chronic hepatitis B or C. The positive effect is even more profound for those with chronic hepatitis B. 

Some might criticize that they have been devised as glucose-lowering medications and are therefore mostly of interest to diabetologists; recently, widespread use of SGLT2I was noted, and several possible mechanisms beyond the glucose lowering effect have been established [13,14,15]. Others probably make a critical comment that the time of observation is not long enough for HCC to develop, suggesting the finding is the result of unadjusted bias. Others might also challenge that there is no time gradient, such that those with longer observation had a lower risk of HCC than those with shorter observation. However, while looking at Table 1, the follow-up period of the study cohort is even longer than the controls. In addition, there are many similarities between the matched cohorts that reflect an effective matching procedure [10]. Such approach seems to be less prone to confounding due to a time lag bias and immortal time bias, as SGLT2I has been introduced as the latest class of drugs and has been used in several patients as an advanced line of therapy. Table 2 reports that all other glucose-lowering medications would protect from HCC, which of course, does make sense as diabetes is a risk factor for HCC, and most patients use one or more of such medications. In addition, the risk is even more decreased in the subgroup of no glucose-lowering medications for SGLT2I users relative to BB users, which is indicative of the oncoprotective effect of SGLT2I among people affected by chronic hepatitis B or C [16]. Furthermore, the protective effect is in a relatively dose-dependent manner, implying our observation is true. 

The pharmacological and pathophysiological background of the study rationale and the hypothesis developed are clear. BB has been shown to be involved in cancer prophylaxis for HCC mainly through a hemodynamic effect [5,6]. Widespread indication of SGLT2I has been established except for glucose-lowering effects. The reasons for SGLT2I being superior to BB might be related to anti-inflammatory, anti-fibrosis, and anti-oxidative effects beyond hemodynamic effects [13,14,15,16]. Interestingly, the risk reduction for HCC among chronic hepatitis is even more dominant for those with chronic hepatitis B, implying a different mechanism of SGLT2I involved in the cancer development of HCC between hepatitis B and C. Although we performed propensity score matching, this can only account for measured variables. In this study, beta-blockers could be used for hypertension and cardiovascular diseases. They might also be used for the treatment of varices or even prior variceal hemorrhage. The latter is obviously associated with a much higher risk of HCC. Either SGLT2 inhibitors reduced HCC risk, or the association between beta-blockers and HCC risk might be possible.

Taken together, our findings provide much more information regarding where to place these results in the context of the already published literature or any trials being conducted in this field. Further studies are necessary to detect deeper insights into the potential patho-physiological and biochemical explanations of the findings. 

## 5. Limitations

This study compares the incidence of HCC in patients with DM coexisting with chronic hepatitis B or C prescribed by SGLT2I or BB. Previous information presented on the validity of codes for diagnoses supports the evidence that these codes are accurate [17,18,19]. The mean follow-up period in this study is 2 years. This seems to be a rather short period for HCC development, and the liver status that enrollees had at the time they were entered into this observational cohort might be a major, obvious limitation. In addition, unfortunately, the lack of information on either disease severity or other biomarkers might make it difficult to interpret our results. Finally, such a strong conclusion can not be made based on this retrospective study using data from a large nationwide database using ICD codes, even with all of its known limitations. 

## 6. Conclusions

This study matched patients using propensity score matching and found that the use of SGLT2I was associated with a significant decrease in HCC occurrence. Although the firm role of SGLT2I cannot be established at this stage, chronic hepatitis B or C patients coexisting with DM should receive intense surveillance for HCC development, which might be an acceptable approach.

## Figures and Tables

**Table 1:** Baseline characteristics among DM patients with HBV/HCV receiving β-blockers or SGLT2is.

Variable	β-Blocker	SGLT2is	*p*-Value
	n (%)/Mean ± SD	n (%)/Mean ± SD	
**All**	7023	7023	
**Sex**			0.8780
Female	2998 (42.69)	3007 (42.82)	
Male	4025 (57.31)	4016 (57.18)	
**Age group (year)**			0.9788
<50	1189 (16.93)	1195 (17.02)	
50–59	2052 (29.22)	2058 (29.30)	
60+	3782 (53.85)	3770 (53.68)	
**Age (year)**	60.39 ± 11.03	60.21 ± 10.97	0.3326
**Comorbidities**			
Hyperlipidemia			0.3174
No	1969 (28.04)	1916 (27.28)	
Yes	5054 (71.96)	5107 (72.72)	
Hypertension			0.7610
No	1887 (26.87)	1903 (27.10)	
Yes	5136 (73.13)	5120 (72.90)	
Obesity			0.8422
No	6813 (97.01)	6817 (97.07)	
Yes	210 (2.99)	206 (2.93)	
Coronary heart disease			0.4661
No	5000 (71.19)	5039 (71.75)	
Yes	2023 (28.81)	1984 (28.25)	
Chronic obstructive pulmonary disease			0.4357
No	6099 (86.84)	6130 (87.28)	
Yes	924 (13.16)	893 (12.72)	
Chronic kidney disease			0.7957
No	6170 (87.85)	6180 (88.00)	
Yes	853 (12.15)	843 (12.00)	
Chronic liver disease and cirrhosis			0.9575
No	2412 (34.34)	2409 (34.30)	
Yes	4611 (65.66)	4614 (65.70)	
Alcohol-related disorders			0.9186
No	6560 (93.41)	6563 (93.45)	
Yes	463 (6.59)	460 (6.55)	
**Medications**			
α-glucosidase inhibitors			0.9277
No	4795 (68.28)	4800 (68.35)	
Yes	2228 (31.72)	2223 (31.65)	
Biguanides			0.1708
No	306 (4.36)	340 (4.84)	
Yes	6717 (95.64)	6683 (95.16)	
Dipeptidyl peptidase-4 inhibitors			0.9044
No	2868 (40.84)	2875 (40.94)	
Yes	4155 (59.16)	4148 (59.06)	
Meglitinides			0.8475
No	5662 (80.62)	5671 (80.75)	
Yes	1361 (19.38)	1352 (19.25)	
Sulphonylureas			0.8910
No	1729 (24.62)	1736 (24.72)	
Yes	5294 (75.38)	5287 (75.28)	
Thiazolidinediones			0.2659
No	4997 (71.15)	4937 (70.30)	
Yes	2026 (28.85)	2086 (29.70)	
Glucagon-like peptide-1 receptor agonists			0.3450
No	6949 (98.95)	6960 (99.10)	
Yes	74 (1.05)	63 (0.90)	
Insulins			0.3482
No	2975 (42.36)	3030 (43.14)	
Yes	4048 (57.64)	3993 (56.86)	
**Follow-up period (year)**	2.05 ± 1.00	2.20 ± 0.83	<0.0001

Abbreviation: DM, diabetes mellitus; HBV, hepatic B virus; HCV, hepatic C virus; SGLT2i, sodium glucose cotransporter 2 inhibitors.

**Table 2:** Risks of HCC associated with SGLT2is in comparison with β-blockers among DM patients with HBV/HCV considering different baseline characteristics.

Variable	IR ^#^		HR (95% CI)	
	Beta Blockers	SGLT2is	Crude	Adjusted ^$^
**All**	19.59	5.11	0.27 (0.21, 0.34) ***	0.27 (0.21, 0.34) ***
**Sex**				
Female	10.71	4.48	0.43 (0.28, 0.66) ***	0.42 (0.27, 0.64) ***
Male	26.43	5.60	0.22 (0.16, 0.29) ***	0.22 (0.16, 0.29) ***
**Age group (year)**				
<50	7.26	0.73	0.10 (0.02, 0.44) **	0.10 (0.02, 0.45) **
50–59	18.61	4.08	0.22 (0.13, 0.37) ***	0.22 (0.13, 0.37) ***
60+	24.52	7.21	0.30 (0.22, 0.40) ***	0.29 (0.22, 0.40) ***
**Comorbidities**				
Hyperlipidemia				
No	29.93	7.91	0.27 (0.19, 0.40) ***	0.28 (0.19, 0.41) ***
Yes	15.87	4.08	0.26 (0.19, 0.36) ***	0.25 (0.18, 0.35) ***
Hypertension				
No	19.25	3.81	0.20 (0.12, 0.35) ***	0.20 (0.11, 0.34) ***
Yes	19.72	5.60	0.29 (0.22, 0.38) ***	0.29 (0.22, 0.38) ***
Obesity				
No	19.84	5.14	0.26 (0.20, 0.34) ***	0.26 (0.20, 0.34) ***
Yes	11.62	4.30	0.39 (0.08, 2.01)	0.28 (0.03, 2.44)
Coronary heart disease				
No	20.88	4.93	0.24 (0.18, 0.33) ***	0.25 (0.18, 0.33) ***
Yes	16.46	5.61	0.34 (0.21, 0.54) ***	0.30 (0.19, 0.48) ***
Chronic obstructive pulmonary disease				
No	20.12	5.24	0.27 (0.20, 0.35) ***	0.27 (0.21, 0.35) ***
Yes	15.95	4.20	0.27 (0.12, 0.59) **	0.25 (0.12, 0.56) ***
Chronic kidney disease				
No	19.72	4.74	0.24 (0.19, 0.32) ***	0.24 (0.19, 0.32) ***
Yes	18.55	8.09	0.45 (0.24, 0.85) *	0.42 (0.22, 0.81) **
Chronic liver disease and cirrhosis				
No	10.07	3.76	0.38 (0.23, 0.64) ***	0.34 (0.20, 0.57) ***
Yes	24.77	5.83	0.24 (0.18, 0.32) ***	0.24 (0.18, 0.32) ***
Alcohol-related disorders				
No	19.10	5.04	0.27 (0.21, 0.35) ***	0.27 (0.21, 0.35) ***
Yes	27.70	6.19	0.24 (0.10, 0.58) **	0.22 (0.09, 0.56) **
**Medications**				
α-glucosidase inhibitors				
No	19.59	4.26	0.22 (0.16, 0.30) ***	0.22 (0.16, 0.31) ***
Yes	19.59	6.85	0.36 (0.25, 0.54) ***	0.35 (0.24, 0.52) ***
Biguanides				
No	13.11	3.04	0.22 (0.05, 1.04)	0.15 (0.03, 0.87) *
Yes	19.88	5.21	0.27 (0.21, 0.34) ***	0.27 (0.21, 0.34) ***
Dipeptidyl peptidase-4 inhibitors				
No	16.47	3.15	0.19 (0.11, 0.30) ***	0.19 (0.11, 0.30) ***
Yes	21.83	6.37	0.30 (0.23, 0.41) ***	0.30 (0.23, 0.41) ***
Meglitinides				
No	19.66	4.73	0.24 (0.18, 0.33) ***	0.24 (0.18, 0.32) ***
Yes	19.28	6.72	0.36 (0.21, 0.60) ***	0.36 (0.21, 0.60) ***
Sulphonylureas				
No	8.44	1.97	0.23 (0.10, 0.52) ***	0.19 (0.08, 0.44) ***
Yes	23.25	6.06	0.27 (0.21, 0.35) ***	0.27 (0.21, 0.35) ***
Thiazolidinediones				
No	18.46	4.69	0.26 (0.19, 0.35) ***	0.25 (0.19, 0.35) ***
Yes	22.43	6.05	0.28 (0.19, 0.43) ***	0.28 (0.19, 0.43) ***
Glucagon-like peptide-1 receptor agonists				
No	19.65	5.16	0.27 (0.21, 0.34) ***	0.27 (0.21, 0.34) ***
Yes	13.89	0.00	NA	NA
Insulins				
No	15.52	3.00	0.19 (0.12, 0.31) ***	0.20 (0.12, 0.32) ***
Yes	22.90	6.72	0.30 (0.23, 0.41) ***	0.30 (0.23, 0.41) ***

Abbreviation: CI, confidence interval; DM, diabetes mellitus; HBV, hepatic B virus; HCC, hepatocellular carcinoma; HCV, hepatic C virus; HR, hazard ratios; IR, incidence rate; SGLT2i, sodium glucose cotransporter 2 inhibitors. *: *p* < 0.05; **: *p* < 0.01; ***: *p* < 0.001. ^#^: per 1000 person-years. ^$^: Multivariate model including all variables listed above.

**Table 3:** Risk of HCC associated with different days’ supply of SGLT2is or β-blockers among DM patients with HBV/HCV.

Variable	Event	Person-Years	IR ^#^	HR (95% CI)	
	N = 361			Crude	Adjusted ^$^
**SGLT2is**					
No (β-blockers)	282	14,393	19.59	1 (Reference)	1 (Reference)
1–532 days	73	6623	11.02	0.52 (0.40, 0.67) ***	0.53 (0.41, 0.68) ***
>532 days	6	8823	0.68	0.04 (0.02, 0.09) ***	0.04 (0.02, 0.08) ***
**β-blockers**					
No (SGLT2is)	79	15,446	5.11	1 (Reference)	1 (Reference)
1–35 days	154	6966	22.11	4.20 (3.20, 5.51) ***	4.20 (3.20, 5.51) ***
>35 days	128	7427	17.24	3.34 (2.52, 4.42) ***	3.36 (2.54, 4.45) ***

Abbreviation: CI, confidence interval; DM, diabetes mellitus; HBV, hepatic B virus; HCC, hepatocellular carcinoma; HCV, hepatic C virus; HR, hazard ratios; IR, incidence rate; SGLT2i, sodium glucose cotransporter 2 inhibitors. ***: *p* < 0.001. ^#^: per 1000 person-years. ^$^: Multivariate model including all variables listed in Table 2.

**Table 4:** Risk of HCC associated with SGLT2is or β-blockers among DM patients stratified by HBV/HCV.

HBV/HCV	SGLT2is	Event	Person-Years	IR ^#^	Crude HR (95% CI)	Adjusted HR ^$^ (95% CI)
HBV	No (β-blockers)	147	9464	15.53	1 (Reference)	1 (Reference)
HBV	Yes	45	11,502	3.91	0.26 (0.18, 0.36) ***	0.25 (0.18, 0.35) ***
HCV	No (β-blockers)	128	5506	23.25	1 (Reference)	1 (Reference)
HCV	Yes	33	4470	7.38	0.32 (0.22, 0.47) ***	0.32 (0.22, 0.46) ***

Abbreviation: CI, confidence interval; DM, diabetes mellitus; HBV, hepatic B virus; HCC, hepatocellular carcinoma; HCV, hepatic C virus; HR, hazard ratios; IR, incidence rate; SGLT2i, sodium glucose cotransporter 2 inhibitors. ***: *p* < 0.001. ^#^: per 1000 person-years. ^$^: Multivariate model including all variables listed in Table 2.

## Data Availability

Data are available upon reasonable request.

## Supplementary Materials

The following supporting information can be downloaded at: https://www.mdpi.com/article/10.3390/cancers15072104/s1, Table S1. Definitions of diseases and medications.
