# Supplementary material for: Sodium-Glucose Cotransporter-2 Inhibitor versus Beta-Blocker Use for Hepatocellular Carcinoma Risk among People with Hepatitis B or C Virus Infection and Diabetes Mellitus
Source: Cancers (Basel). 2023 Mar 31;15(7):2104. doi: 10.3390/cancers15072104 (PMC10093089; doi:10.3390/cancers15072104)
Supplement: Supplementary file 1 [file cancers-15-02104-s001.zip › cancers-2236786-supplementary.pdf]

Sodium-glucose cotransporter-2 inhibitors versus beta blockers use for hepatocellular carcinoma risk among people with hepatitis B or C virus infection and diabetes mellitus

Wei-Syun Hu[1,2],MD, PhD; Cheng-Li Lin[3], MS;

Table S1. Definitions of diseases and medications

Algorithm: 1 hospitalization or at least 3 outpatient visits		
**Disease**	**ICD-9-CM**	**ICD-10-CM**
Diabetes mellitus	250	E08-E13
Hepatic B virus	070.2, 070.3, V02.61	B16.0-B16.2, B16.9, B18.0, B18.1, B19.10, B19.11, Z22.51
Hepatic C virus	070.41, 070.44, 070.51, 070.54, V02.62	B17.10, B17.11, B18.2, B19.20, B19.21, Z22.52
Hepatocellular carcinoma#	155, 197.7	C22, C78.7, C7B.02
Hyperlipidemia	272.0-272.4	E78.0-E78.5
Hypertension	401-405	I10-I15
Obesity	278	E65-E68
Coronary heart disease	410-414	I20-I25
Chronic obstructive pulmonary disease	491, 492, 496	J41-J44
Chronic kidney disease	585	N18
Chronic liver disease and cirrhosis	571	K70, K73, K74, K75.4, K75.81, K76.0, K76.89, K76.9
Alcohol-related disorders	291, 303, 305.0, 571.0-571.3, 790.3, V11.3, V79.1	F10, K70, R78.0, Z65.8
#: at least one catastrophic illness registry		

Algorithm: At least 1 medication claim	
**Medication**	**ATC code**
Sodium glucose cotransporter 2 inhibitors	A10BK01-A10BK04, A10BD15, A10BD19-A10BD21, A10BD24
β-blockers	C07
α-glucosidase inhibitors	A10BF01, A10BF02
Biguanides	A10BA02, A10BA03, A10BD, A10BD02, A10BD03, A10BD05, A10BD07-A10BD11, A10BD13, A10BD15, A10BD19- A10BD21, A10BD24
Dipeptidyl peptidase-4 inhibitors	A10BH01-A10BH05, A10BD07, A10BD08-A10BD11, A10BD13, A10BD19, A10BD21, A10BD24

Meglitinides	A10BX02, A10BX03, A10BX08, A10BD
Sulphonylureas	A10BB01, A10BB02, A10BB03, A10BB05, A10BB07, A10BB08, A10BB09, A10BB12, A10BD02
Thiazolidinediones	A10BG02, A10BG03, A10BD05, A10BD09
Glucagon-like peptide-1 receptor agonists	A10BJ01, A10BJ02, A10BJ03, A10BJ05, A10BJ06, A10AE54
Insulins	A10AB01, A10AB04, A10AB05, A10AB06, A10AC01, A10AD01, A10AD05, A10AE04, A10AE05, A10AE06, A10AE54, N05BA91
